# Shade triggers posttranscriptional PHYTOCHROME-INTERACTING FACTOR-dependent increases in H3K4 trimethylation

**DOI:** 10.1093/plphys/kiac282

**Published:** 2022-06-08

**Authors:** Robert H Calderon, Jutta Dalton, Yu Zhang, Peter H Quail

**Affiliations:** Department of Plant and Microbial Biology, University of California, Berkeley, California, 94720, USA; Plant Gene Expression Center, Agriculture Research Service, US Department of Agriculture, Albany, California, 94710, USA; Department of Plant Physiology, Umeå Plant Science Centre, Umeå University, Umeå, 901 87, Sweden; Department of Plant and Microbial Biology, University of California, Berkeley, California, 94720, USA; Plant Gene Expression Center, Agriculture Research Service, US Department of Agriculture, Albany, California, 94710, USA; Department of Plant and Microbial Biology, University of California, Berkeley, California, 94720, USA; Plant Gene Expression Center, Agriculture Research Service, US Department of Agriculture, Albany, California, 94710, USA; US Department of Energy, Joint Genome Institute, Lawrence Berkeley National Laboratory, Berkeley, California, 94720, USA; Department of Plant and Microbial Biology, University of California, Berkeley, California, 94720, USA; Plant Gene Expression Center, Agriculture Research Service, US Department of Agriculture, Albany, California, 94710, USA

## Abstract

The phytochrome (phy)-PHYTOCHROME-INTERACTING FACTOR (PIF) sensory module perceives and transduces light signals to direct target genes (DTGs), which then drive the adaptational responses in plant growth and development appropriate to the prevailing environment. These signals include the first exposure of etiolated seedlings to sunlight upon emergence from subterranean darkness and the change in color of the light that is filtered through, or reflected from, neighboring vegetation (“shade”). Previously, we identified three broad categories of rapidly signal-responsive genes: those repressed by light and conversely induced by shade; those repressed by light, but subsequently unresponsive to shade; and those responsive to shade only. Here, we investigate the potential role of epigenetic chromatin modifications in regulating these contrasting patterns of phy-PIF module-induced expression of DTGs in Arabidopsis (*Arabidopsis thaliana*). Using RNA-seq and ChIP-seq to determine time-resolved profiling of transcript and histone 3 lysine 4 trimethylation (H3K4me3) levels, respectively, we show that, whereas the initial dark-to-light transition triggers a rapid, apparently temporally coincident decline of both parameters, the light-to-shade transition induces similarly rapid increases in transcript levels that precede increases in H3K4me3 levels. Together with other recent findings, these data raise the possibility that, rather than being causal in the shade-induced expression changes, H3K4me3 may function to buffer the rapidly fluctuating shade/light switching that is intrinsic to vegetational canopies under natural sunlight conditions.

## Teaser

The shade-induced transcription of phytochrome-regulated genes precedes increases in the accumulation of the chromatin-modifying mark.

## Introduction

All organisms must perceive, process, and react to environmental cues in order to survive and pass their genetic material onto the next generation. Land plants in particular, given their sessile lifestyle, must quickly perceive these environmental signals and respond accordingly. One particularly well-studied plant signaling system is the phytochrome (phy) family of photoreceptors (phyA to phyE in Arabidopsis (*Arabidopsis thaliana*)), a set of red (R) and far-red (FR) light-absorbing chromoproteins that transduce light signals into large-scale changes in gene expression (Tepperman et al., 2001). Upon absorption of R light, the inactive form of the phy molecule (Pr) is photoconverted into the active form (Pfr) which quickly translocates from the cytoplasm to the nucleus, initiating downstream developmental programs, directed by these expression changes (Sakamoto and Nagatani, 1996).

Experimental evidence indicates that a critical link between these downstream programs and the phy molecules is a subfamily of eight basic helix-loop-helix (bHLH) transcription factors called PHY-INTERACTING FACTORS (PIFs; Ni et al., 1998; Huq and Quail, 2002; Monte et al., 2004; Leivar and Quail, 2011; Pham et al., 2018). The PIFs, in particular PIF1, PIF3, PIF4, and PIF5 (called the PIF quartet), form a set of partially functionally redundant proteins that bind to a consensus sequence in the upstream region of target genes, regulating their transcriptional output (Leivar et al., 2009). The PIF quartet has been shown to physically interact specifically with the Pfr form of phyB, which subsequently induces phosphorylation, ubiquitination, and degradation of the transcription factor (Ni et al., 2013, 2014, 2017), thereby triggering global changes in target gene expression (Leivar et al., 2009; Leivar and Quail, 2011; Pham et al., 2018). In addition to the PIF quartet, PIF6 and PIF7 have also been shown to function in phyB signaling, with PIF7, in particular, serving as a key regulator of auxin biosynthesis during the shade-avoidance response (Khanna et al., 2004; Leivar et al., 2008a, 2008b; Li et al., 2012). The integration of several genome-wide analyses of PIF-binding and PIF-mediated transcriptional regulation (Leivar et al., 2009, 2012; Hornitschek et al., 2012; Oh et al., 2012) has led to the discovery of over 300 direct target genes (DTGs) that are directly, transcriptionally regulated by PIFs (Zhang et al., 2013; Pfeiffer et al., 2014).

The relative abundance of the Pfr and Pr forms of the phyB molecule, and by extension the accumulation and activity of the PIFs, is determined by the ratio of R to FR light in the immediate environment. The active Pfr form is favored under white-light (WL) illumination where the R/FR ratio is high, whereas the inactive Pr form is favored in the dark and in conditions where the R/FR ratio is low, such as under vegetative shading (Quail et al., 1995). As a consequence of the photoreversible nature of the phyB molecule, PIF accumulation and activity is high in darkness and in the shade. The transcriptional responses of many PIF DTGs, however, do not exhibit a photoreversible pattern (Leivar et al., 2012).

In a previous study, we were able to categorize the transcriptional responses of PIF DTGs into three distinct patterns: those that respond during the transition from the etiolated dark-grown state to R, those that respond during the transition from WL into simulated shade, or those that respond during both transitions (Leivar et al., 2012). The differential responsiveness of these three broad sets of PIF DTGs indicates that PIF abundance is not the sole determinant of PIF DTG expression. Core components of the plant circadian oscillator have been implicated in modulating some of these changes in gene expression (Martín et al., 2018; Zhang et al., 2020). Most recently, changes in the chromatin environment have been shown to be directly involved in triggering shade-induced transcription (Willige et al., 2021).

One form of chromatin remodeling that can modulate the transcriptional output of light-regulated genes involves the enzymatic modification of histones (Fisher and Franklin, 2011; Perrella and Kaiserli, 2016; Bourbousse et al., 2019; Martínez-García and Moreno-Romero, 2020). Methylation, acetylation, and/or ubiquitination of histones have all been shown to regulate transcription of light-regulated genes (Charron et al., 2009; Bourbousse et al., 2012; Liu et al., 2013). Unique histone modification patterns at the promoters of individual PIF DTGs have the potential to underly the differential responsiveness of PIF DTGs under different environmental conditions. The accumulation of one particular mark, histone 3 lysine 4 trimethylation (H3K4me3), at the transcriptional start site (TSS) of genes has long been known to strongly correlate with transcriptional activity of those genes (Bernstein et al., 2002), but the biological function of this mark remains relatively less-well defined (Fiorucci et al., 2019). Proposed roles include facilitating transcriptional elongation (Ding et al., 2012) or serving as “transcriptional memory” (Liu et al., 2014).

Here, we have refined the list of PIF DTGs by integrating previously published chromatin immunoprecipitation (ChIP) binding and RNA-seq data for the PIF quartet, with additional RNA-seq data from both wild-type (WT) and a mutant lacking six of the PIFs (PIF1, 3, 4, 5, 6, and 7). Using this system, we have explored the potential role of the epigenetic mark H3K4me3 in mediating the observed differential patterns of expression of PIF DTGs. Our data suggest a possible functional role for H3K4me3 in stabilizing the expression levels of DTGs in established green plants, against the rapidly switching light/shade transitions that occur naturally in leaf canopies.

## Results

### Characterization of the *pifqpif6pif7* sextuple mutant

**Figure 1:**
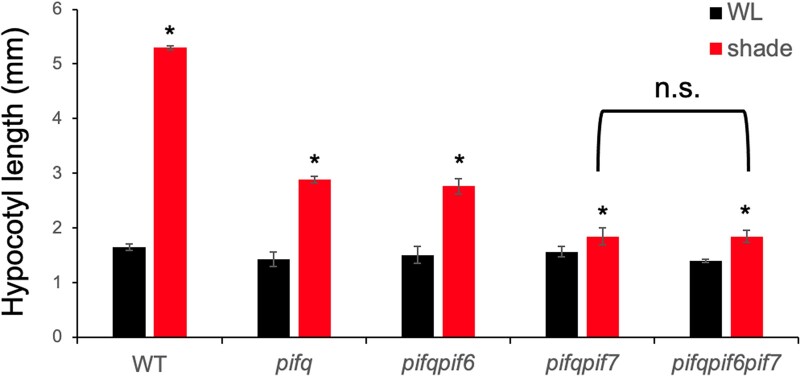
Phenotypic analysis of higher-order *pif* mutants in response to simulated shade. Hypocotyl lengths of WT, *pifq*, *pifqpif6, pifqpif7*, and *pifqpif6pif7* mutants grown for 6 days in WL or 2 days in WL followed by 4 days in simulated shade (shade). Data represent the mean and se from three biological replicates of 30 seedlings per genotype. Asterisks indicate that the hypocotyl lengths of shade-treated seedlings are statistically significantly different from the corresponding WL controls by Student’s *t* test (*P* < 0.05). n.s. indicates “not significantly different” (*P* > 0.99).

The *pif1pif3pif4pif5* quadruple mutant (hereafter *pifq*) displays a constitutively photomorphogenic phenotype when grown in darkness, indicating that these four PIFs are necessary and sufficient to control de-etiolation in response to R (Leivar et al., 2008a, 2008b, 2009). The *pifq* mutant does not, however, exhibit a complete lack of responsiveness to simulated shade (Figure 1), supporting the hypothesis that additional factors are required for the complete shade avoidance response (Leivar et al., 2012). PIF7 has been implicated in playing a major role in regulating this process (Li et al., 2012; de Wit et al., 2015; Mizuno et al., 2015) with the quintuple *pifqpif7* mutant reported to show no statistically significant shade avoidance response (Zhang et al., 2020).

However, when we measured the shade avoidance response in the *pifqpif7* mutant under slightly different conditions to Zhang et al. (2020), we were still able to detect a small, yet statistically significant (*P* < 0.05) residual shade avoidance response (Figure 1). A possible reason for this small difference is that the results presented here were obtained on 2-day-old seedlings exposed to simulated shade, whereas our previous experiments were performed on 3-day-old seedlings exposed to simulated shade. Alternatively, this minor residual shade-avoidance response observed under our conditions could be due to the presence of yet other members of the PIF-subfamily, such as PIF8 or PIL1 (PIF2; Leivar and Quail, 2011; Pham et al., 2018), or to other light-responsive transcription factors. Nevertheless, we then tested whether PIF6 might be responsible for this residual response by generating a sextuple *pifqpif6pif7 (pifS)* mutant and measuring its hypocotyl length in response to simulated shade. This sextuple mutant displayed significantly shorter hypocotyls than the WT in response to shade, but no significant decrease relative to the *pifqpif7* quintuple mutant (Figure 1). These results suggest that PIF6 plays no significant role in mediating the shade-avoidance response, consistent with its proposed role in seed dormancy and development (Penfield et al., 2010).

### Generation of a high-confidence list of PIF DTGs and subcategorization into E, ES, and S classes

Many PIF DTGs have been previously observed to be upregulated in the presence of the PIFs while others are downregulated. For the purposes of this study, we focused only on PIF-induced genes (i.e. those genes which appear to require the PIFs for high levels of transcription) because PIFs have been shown to have intrinsic activating activity (Huq et al., 2004; Al-Sady et al., 2008; de Lucas et al., 2008; Dalton et al., 2016).

In brief, we first integrated the data from a previously published RNA-seq experiment on dark-grown seedlings exposed to 1 h of R light (Pfeiffer et al, 2014) with an RNA-seq time-course experiment of WL-grown seedlings exposed to 3 h of simulated shade (shade light). We then combined previously published RNA-seq data from the *pifq* mutant grown in darkness (Pfeiffer et al., 2014), with RNA-seq data that were obtained using the *pifqpif6pif7* mutant (*pifS*) grown in WL and exposed to 3-h shade light. Lastly, we used previously published data to identify those genes whose promoters were found to be bound by PIF1, PIF3, PIF4, PIF5, and/or PIF7 (no genome-wide binding data are available for PIF6; Hornitschek et al., 2012; Oh et al., 2012; Zhang et al., 2013; Pfeiffer et al., 2014; Chung et al., 2020). By selecting only the genes that met all three of our criteria (light-responsiveness, PIF dependence and PIF binding), we obtained 169 candidate PIF-induced, red-light repressed, and/or shade-light-induced DTGs (Table 1).

**Table 1:** List of all candidate PIF DTGs and categorization into etiolation (E), shade (S), or etiolation and shade (ES)-responsive genes

Locus	Name	R60^a^	*pifQ* ^b^	FR30^c^	FR60	FR120	FR180	*pS* ^d^	PIF bound^e^	Original class^f^	New class^g^	Group^h^	Pfeiffer class^i^	Known PIF DTG	ANOM^j^
AT5G02260	EXP9	−1.31	−2.99	–	–	–	–	–	157	E	E	1	Ind	Yes	N/A
AT5G02580	At5g02580	−2.99	−1.39	–	–	–	–	–	13457	E	E	1	Ind	Yes	N/A
AT5G67020	At5g67020	−2.28	−1.21	–	–	–	–	–	7	E	E	1		Yes	N/A
AT5G02190	PCS1	−2.27	−1.19	–	–	–	–	–	57	E	E	1	Ind	Yes	N/A
AT1G07090	LSH6	−1.09	−1.14	–	–	–	–	–	13457	E	E	1	Ind	Yes	N/A
AT1G67265	DVL3	−4.26	−2.16	–	–	–	–	–	1345	E	E	1	Ind	Yes	N/A
AT1G60060	At1g60060	−2.14	−2.03	–	–	–	–	–	4	E	E	1		Yes	N/A
AT5G15830	BZIP3	1.18	−1.78	–	–	–	–	–	5	E	E	1		Yes	N/A
AT4G37740	GRF2	−2.01	−1.66	–	–	–	–	–	14	E	E	1	Ind	Yes	N/A
AT5G50175	At5g50175	−1.68	−1.65	–	–	–	–	–	14	E	E	1	Ind	Yes	N/A
AT4G36010	At4g36010	−2.30	−1.64	–	–	–	–	–	134	E	E	1	Ind	Yes	N/A
AT4G10020	HSD5	−1.23	−1.64	–	–	–	–	–	14	E	E	1	Ind	Yes	N/A
AT3G25730	EDF3	−2.91	−1.55	–	–	–	–	–	3	E	E	1		Yes	N/A
AT3G28340	GATL10	−1.00	−1.48	–	–	–	–	–	15	E	E	1	Ind	Yes	N/A
AT1G58410	At1g58410	−1.35	−1.28	–	–	–	–	–	14	E	E	1		Yes	N/A
AT3G53200	MYB27	−2.40	−1.07	–	–	–	–	–	14	E	E	1	Ind	Yes	N/A
AT5G53980	ATHB52	−4.07	−1.01	–	–	–	–	–	35	E	E	1	Ind	Yes	N/A
AT2G42870	PAR1	−2.31	–	–	–	–	2.14	−2.34	13457	S	ES	2		Yes	N/A
AT3G59900	ARGOS	−1.01	–	–	1.62	1.90	1.84	−2.13	14	S	ES	2		Yes	N/A
AT2G44910	ATHB-4	−2.10	–	3.95	1.83	1.27	–	−1.50	13457	S	ES	2		Yes	N/A
AT5G28300	GT2L	−2.17	–	–	–	***	1.19	−1.47	13457	S	ES	2		Yes	N/A
AT1G13260	RAV1	−2.51	–	–	–	1.22	–	−1.32	1345	S	ES	2		Yes	N/A
AT5G44260	TZF5	−3.69	–	–	–	2.60	2.85	−3.01	7	S	ES	2		Yes	N/A
AT5G02760	APD7	−1.70	–	–	3.23	3.53	2.95	−2.50	45	S	ES	2	Rep	Yes	N/A
AT5G62280	At5g62280	−1.96	–	–	2.70	3.11	2.74	−2.34	7	S	ES	2		Yes	N/A
AT5G46330	FLS2	−1.52	–	–	–	–	1.62	−1.74	57	S	ES	2		Yes	N/A
AT2G44080	ARL	−2.04	–	–	–	1.68	1.46	−1.41	45	S	ES	2		Yes	N/A
AT3G60390	HAT3	−1.51	–	1.42	1.18	–	–	–	1345	S	ES	3		Yes	N/A
AT5G25190	ESE3	−1.83	–	–	1.24	2.13	***	–	1345	S	ES	3		Yes	N/A
AT1G25560	EDF1	−1.64	–	–	–	1.25	1.05	–	145	S	ES	3		**No**	N/A
AT3G60520	At3g60520	−1.50	–	–	–	1.49	–	–	45	S	ES	3		Yes	N/A
AT4G28240	BGL1	−1.45	–	–	***	1.24	1.04	–	457	S	ES	3		Yes	N/A
AT2G45210	SAUR36	−1.19	−1.25	–	–	***	1.05	–	15	E	ES	1	Ind	Yes	N/A
AT2G43060	IBH1	−1.56	−1.44	–	–	1.04	–	–	13457	ES	ES	1 & 3	Ind	Yes	N/A
AT5G02540	At5g02540	−1.63	−1.49	–	2.50	5.38	6.26	−5.74	13457	ES	ES	1 & 2	Ind	Yes	N/A
AT3G15540	IAA19	−1.46	−1.63	–	3.31	3.37	2.20	−2.62	1345	ES	ES	1 & 2	Ind	Yes	N/A
AT3G21330	At3g21330	−3.11	−1.65	2.52	4.25	4.00	3.90	−3.52	1345	ES	ES	1 & 2	Ind	Yes	N/A
AT5G63650	SNRK2.5	−1.75	−1.68	–	–	1.78	2.30	−2.67	1345	ES	ES	1 & 2	Ind	Yes	N/A
AT5G07010	ST2A	−2.97	−1.71	–	–	–	2.06	−2.87	13457	ES	ES	1 & 2	Ind	Yes	N/A
AT5G01790	At5g01790	−1.41	−1.74	–	–	1.62	1.69	−1.56	145	ES	ES	1 & 2	Ind	Yes	N/A
AT1G10550	XTH33	−1.05	−1.75	–	–	1.21	***	−1.25	13457	ES	ES	1 & 2	Ind	Yes	N/A
AT3G61830	ARF18	−1.63	−1.78	–	–	–	1.04	−1.08	3	ES	ES	1 & 2	Ind	Yes	N/A
AT4G16780	ATHB-2	−2.97	−1.97	3.69	3.03	2.91	2.85	−2.69	13457	ES	ES	1 & 2	Ind	Yes	N/A
AT4G35720	At4g35720	−2.68	−2.27	–	–	1.67	1.38	−1.95	1345	ES	ES	1 & 2	Ind	Yes	N/A
AT4G14130	XTR7	−2.50	−2.41	–	–	2.17	3.82	−3.98	13457	ES	ES	1 & 2	Ind	Yes	N/A
AT4G32280	IAA29	−2.47	−2.56	–	4.51	4.72	4.25	−5.40	1345	ES	ES	1 & 2	Ind	Yes	N/A
AT5G65800	ACS5	−2.04	−2.73	–	2.84	–	–	–	145	ES	ES	1 & 2	Ind	Yes	N/A
AT4G31380	FLP1	−2.18	−2.99	–	3.00	3.82	3.22	−4.20	1457	ES	ES	1 & 2		Yes	N/A
AT2G46970	PIL1	−4.26	−5.56	2.35	2.95	2.94	3.25	−4.62	13457	ES	ES	1 & 2	Ind	Yes	N/A
AT5G05965	At5g05965	−1.97	−1.20	–	–	***	–	−1.62	1345	E	ES	1	Ind	Yes	N/A
AT5G09970	CYP78A7	***	–	–	1.31	1.78	1.01	−1.98	1	S	ES	2		Yes	N/A
AT1G21050	At1g21050	***	–	–	1.49	1.49	1.58	−1.43	1357	S	ES	2		Yes	N/A
AT5G59010	BSK1	***	–	–	–	1.30	***	−1.28	145	S	ES	2		Yes	N/A
AT3G61460	BRH1	***	–	–	1.35	1.39	1.29	−1.21	13457	S	ES	2		Yes	N/A
AT1G21830	At1g21830	***	–	–	1.13	1.10	***	−1.05	1345	S	ES	2		Yes	N/A
AT3G50340	At3g50340	***	–	–	2.48	2.17	1.41	−1.32	5	S	ES	2		Yes	N/A
AT1G54120	At1g54120	***	–	–	1.58	–	–	–	15	S	ES	3		**No**	N/A
AT4G22780	ACR7	***	–	–	1.08	–	–	–	145	S	ES	3		Yes	N/A
AT2G28400	At2g28400	***	–	–	–	1.15	–	–	3	S	ES	3		Yes	N/A
AT4G25260	PMEI7	***	−2.16	–	–	1.47	1.25	−1.62	145	S	ES	2	Ind	Yes	N/A
AT5G46240	KAT1	***	–	1.55	2.15	1.77	1.62	−1.77	14	S	ES	2		Yes	N/A
AT5G18030	SAUR21	***	–	2.63	3.37	2.46	2.10	−1.53	357	S	ES	2		Yes	N/A
AT4G38860	SAUR16	***	–	–	1.13	–	–	–	135	S	ES	3		Yes	N/A
AT1G02400	GA2OX6	***	–	–	–	1.89	–	–	1345	S	ES	3		Yes	N/A
AT3G05640	EGR1	***	–	–	–	1.17	–	–	457	S	ES	3		**No**	N/A
AT3G62070	At3g62070	***	−1.33	–	1.53	1.05	–	–	5	S	ES	3		**No**	N/A
AT1G29430	SAUR62	***	–	1.18	2.22	1.02	–	–	5	S	ES	3		**No**	N/A
AT1G75450	CKX5	***	−1.66	–	–	1.68	2.04	−1.96	13457	S	ES	2	Ind	Yes	N/A
AT5G18060	SAUR23	***	−1.73	–	3.00	2.53	2.14	−2.18	157	S	ES	2	Ind	Yes	N/A
AT4G37770	ACS8	***	−2.29	–	4.62	4.64	4.96	−5.52	7	S	ES	2		Yes	N/A
AT4G13790	SAUR25	***	−3.24	–	4.15	–	–	–	15	S	ES	3	Ind	Yes	N/A
AT3G62090	PIF6	***	−3.57	–	–	3.99	3.85	−6.90	13457	S	ES	2	Ind	Yes	N/A
AT3G12820	MYB10	***	−4.10	–	–	2.34	2.97	−2.25	4	S	ES	2		Yes	N/A
AT3G21320	At3g21320	–	–	–	6.54	7.28	7.16	−7.75	13457	S	S	2		Yes	N/A
AT5G22500	FAR1	–	–	–	–	2.31	3.50	−3.44	145	S	S	2		Yes	N/A
AT4G28720	YUC8	–	–	1.36	2.19	2.36	2.55	−2.88	13457	S	S	2		Yes	N/A
AT1G04180	YUC9	–	–	4.42	4.24	3.25	2.68	−2.71	1345	S	S	2		Yes	N/A
AT5G18050	SAUR22	–	–	–	3.89	3.34	3.01	−2.58	157	S	S	2		Yes	N/A
AT1G02350	At1g02350	–	–	–	3.31	2.81	2.98	−2.20	13457	S	S	2		Yes	N/A
AT5G66080	APD9	–	–	–	1.26	1.47	1.62	−1.88	1457	S	S	2		Yes	N/A
AT3G23030	IAA2	–	–	1.29	2.32	2.29	1.98	−1.73	1345	S	S	2		Yes	N/A
AT5G47370	HAT2	–	–	1.40	3.40	2.34	1.62	−1.54	1345	S	S	2		Yes	N/A
AT4G14560	IAA1	–	–	–	3.10	2.19	1.89	−1.54	134	S	S	2		Yes	N/A
AT5G25460	DGR2	–	–	–	–	***	1.12	−1.25	1357	S	S	2		Yes	N/A
AT1G36940	At1g36940	–	–	–	–	1.06	***	−1.20	15	S	S	2		Yes	N/A
AT3G23050	IAA7	–	–	–	–	–	1.13	−1.01	1345	S	S	2		Yes	N/A
AT2G23170	GH3.3	–	–	–	2.30	3.57	3.29	−3.14	3	S	S	2		Yes	N/A
AT5G12050	BG1	–	–	2.93	3.56	3.36	2.87	−2.28	57	S	S	2		Yes	N/A
AT1G76610	At1g76610	–	–	–	2.44	2.51	2.04	−1.96	7	S	S	2		Yes	N/A
AT1G29465	At1g29465	–	–	–	1.77	2.75	2.50	−1.63	5	S	S	2		**No**	N/A
AT1G75500	WAT1	–	–	–	***	1.39	1.34	−1.42	7	S	S	2		Yes	N/A
AT1G21980	PIP5K1	–	–	–	***	***	1.24	−1.28	3	S	S	2		Yes	N/A
AT1G31880	BRX	–	–	–	1.32	1.52	1.09	−1.10	4	S	S	2		Yes	N/A
AT4G39800	MIPS1	–	–	–	–	1.41	1.28	−1.03	5	S	S	2		Yes	N/A
AT1G67900	At1g67900	–	–	–	2.61	2.06	1.36	−1.03	7	S	S	2		Yes	N/A
AT5G16023	DVL1	–	–	–	2.48	–	–	–	1345	S	S	3		Yes	N/A
AT5G39860	PRE1	–	–	–	2.42	–	–	–	157	S	S	3		Yes	N/A
AT4G34760	SAUR50	–	–	–	1.08	***	–	–	145	S	S	3		Yes	N/A
AT1G49780	PUB26	–	–	***	1.06	***	–	–	13457	S	S	3		**No**	N/A
AT4G32290	At4g32290	–	–	–	1.04	***	***	–	15	S	S	3		**No**	N/A
AT5G43890	YUC5	–	–	2.14	–	–	–	–	145	S	S	3		Yes	N/A
AT3G62100	IAA30	–	–	–	2.60	2.32	–	–	134	S	S	3		**No**	N/A
AT4G37390	GH3.2	–	–	–	1.29	2.04	–	–	1345	S	S	3	Rep	Yes	N/A
AT1G75490	At1g75490	–	–	–	–	1.99	–	–	145	S	S	3	Rep	Yes	N/A
AT4G24275	At4g24275	–	–	–	1.11	1.79	1.04	–	14	S	S	3		**No**	N/A
AT4G27280	CMI1	–	–	–	1.84	1.61	–	–	1	S	S	3		Yes	N/A
AT4G27310	BBX28	–	–	–	–	1.54	1.66	–	13457	S	S	3		Yes	N/A
AT5G59220	HAI1	–	–	–	–	1.50	–	–	1,345	S	S	3	Ind	Yes	N/A
AT1G60190	PUB19	–	–	–	–	1.45	–	–	13457	S	S	3		Yes	N/A
AT2G40610	EXP8	–	–	–	–	1.44	–	–	1345	S	S	3		Yes	N/A
AT5G54510	GH3.6	–	–	–	***	1.27	1.04	–	135	S	S	3		Yes	N/A
AT2G45420	LBD18	–	–	–	–	1.24	–	–	1	S	S	3		**No**	N/A
AT5G60840	At5g60840	–	–	–	***	1.12	***	–	13457	S	S	3		Yes	N/A
AT4G09890	At4g09890	–	–	–	1.30	1.12	–	–	145	S	S	3		Yes	N/A
AT5G62220	GT18	–	–	–	–	1.09	–	–	15	S	S	3		Yes	N/A
AT3G19380	PUB25	–	–	–	***	1.08	–	–	1345	S	S	3		Yes	N/A
AT3G44310	NIT1	–	–	–	–	1.07	1.25	–	13457	S	S	3		Yes	N/A
AT5G16200	At5g16200	–	–	–	–	1.03	–	–	15	S	S	3		**No**	N/A
AT1G21910	DREB26	–	–	–	–	1.01	***	–	15	S	S	3		Yes	N/A
AT3G03850	SAUR26	–	–	–	3.17	–	–	–	5	S	S	3		Yes	N/A
AT3G03840	SAUR27	–	–	–	2.89	–	–	–	5	S	S	3		Yes	N/A
AT2G18010	SAUR10	–	–	–	4.65	3.72	3.53	–	5	S	S	3		Yes	N/A
AT3G03830	SAUR28	–	–	–	4.00	2.87	–	–	5	S	S	3		Yes	N/A
AT4G34770	SAUR1	–	–	–	2.31	2.13	–	–	5	S	S	3		Yes	N/A
AT1G29460	SAUR65	–	–	–	2.83	1.99	1.58	–	5	S	S	3		Yes	N/A
AT1G29500	SAUR66	–	–	–	2.53	1.91	–	–	35	S	S	3		Yes	N/A
AT3G55840	At3g55840	–	–	–	–	1.83	–	–	5	S	S	3		Yes	N/A
AT5G18020	SAUR20	–	–	1.98	2.37	1.82	1.42	–	357	S	S	3		Yes	N/A
AT1G29440	SAUR63	–	–	–	2.31	1.73	–	–	5	S	S	3		Yes	N/A
AT1G29450	SAUR64	–	–	–	2.51	1.70	–	–	5	S	S	3		Yes	N/A
AT1G52565	At1g52565	–	–	–	–	1.68	–	–	5	S	S	3		**No**	N/A
AT1G69160	WIP1	–	–	–	1.41	1.26	1.20	–	5	S	S	3	Rep	Yes	N/A
AT5G18010	SAUR19	–	−1.10	–	3.28	2.68	–	−2.39	145	S	S	2	Ind	Yes	N/A
AT3G50350	At3g50350	–	−1.16	–	1.12	1.99	1.15	–	5	S	S	3		**No**	N/A
AT3G50800	At3g50800	–	−1.20	1.97	2.47	2.77	2.25	−2.03	13457	S	S	2	Ind	Yes	N/A
AT1G04240	IAA3	–	−1.22	–	1.66	–	–	–	1457	S	S	3	Ind	Yes	N/A
AT1G76240	At1g76240	–	−1.26	–	–	1.01	1.04	−1.01	15	S	S	2	Ind	Yes	N/A
AT1G18400	BEE1	–	−1.30	–	2.50	2.35	1.80	−1.60	1345	S	S	2	Ind	Yes	N/A
AT5G66580	At5g66580	–	−1.32	2.35	3.63	2.60	1.67	–	13457	S	S	3	Ind	Yes	N/A
AT3G28857	PRE5	–	−1.79	–	3.28	–	–	–	145	S	S	3	Ind	Yes	N/A
AT2G14960	GH3.1	–	−1.90	–	–	1.41	–	–	13	S	S	3	Ind	Yes	N/A
AT1G06080	ADS1	–	−2.01	–	–	3.18	3.94	−4.13	4	S	S	2		Yes	N/A
AT5G66590	At5g66590	–	−2.06	–	1.40	1.27	1.03	−1.05	13457	S	S	2	Ind	Yes	N/A
AT1G16850	At1g16850	–	−2.82	–	–	1.03	–	–	135	S	S	3	Ind	Yes	N/A
AT2G31980	CYS2	−1.68	−3.00	–	–	–	–	–	37	E	ANOM	1		N/A	High in WL
AT1G10560	PUB18	−1.51	−2.06	–	–	–	–	–	13457	E	ANOM	1	Ind	N/A	High in WL
AT1G11960	At1g11960	−1.32	−1.21	–	–	–	–	–	37	E	ANOM	1		N/A	High in WL
AT3G61680	PLIP1	−1.32	−1.05	–	–	–	–	–	7	E	ANOM	1		N/A	High in WL
AT1G77200	At1g77200	−2.31	−1.80	–	–	1.29	–	–	1345	ES	ANOM	1 & 3	Ind	N/A	High in WL
AT1G36060	TG	−1.04	−1.29	–	–	–	–	–	5	E	ANOM	1	Ind	N/A	High in WL
AT1G02340	HFR1	–	−3.82	2.56	3.05	3.41	3.44	−3.18	13457	S	ANOM	2	Ind	N/A	R-induced
AT3G54200	NHL39	–	−1.04	–	–	2.04	1.73	−1.77	1345	S	ANOM	2		N/A	artifactual
AT1G69570	CDF5	–	–	–	2.21	2.32	2.08	−1.55	13457	S	ANOM	2		N/A	artifactual
AT4G01680	MYB55	–	–	–	–	1.13	1.28	−1.22	145	S	ANOM	2		N/A	R-induced
AT1G18710	MYB47	–	–	–	1.35	1.36	–	−1.02	1	S	ANOM	2		N/A	R-induced
AT2G33380	RD20	–	−3.08	–	–	2.66	2.39	−2.24	34	S	ANOM	2	Ind	N/A	R-induced
AT1G80130	At1g80130	–	–	–	–	1.60	2.26	−2.04	5	S	ANOM	2		N/A	R-induced
AT5G54470	BBX29	–	–	–	2.69	2.81	2.88	–	1345	S	ANOM	3	Ind	N/A	R-induced
AT1G09350	GOLS3	–	–	–	1.91	2.10	–	–	13	S	ANOM	3		N/A	R-induced
AT5G66110	HIPP27	–	−1.15	–	–	1.65	1.67	–	1	S	ANOM	3	Ind	N/A	low
AT3G16800	EGR3	–	–	–	1.07	1.38	1.49	–	1457	S	ANOM	3		N/A	R-induced
AT1G73480	MAGL4	–	–	–	–	1.37	–	–	145	S	ANOM	3		N/A	R-induced
AT3G29575	AFP3	–	−1.45	–	–	1.33	–	–	^13^457	S	ANOM	3	Ind	N/A	R-induced
AT3G22830	HSFA6B	–	−2.29	–	–	1.19	–	–	145	S	ANOM	3		N/A	R-induced
AT3G57540	REM4.1	–	–	–	–	1.08	–	–	1	S	ANOM	3		N/A	R-induced
AT2G29440	GST24	–	−2.56	–	–	1.04	–	–	1	S	ANOM	3	Ind	N/A	R-induced
AT1G78440	GA2OX1	–	–	–	2.22	2.38	2.53	–	57	S	ANOM	3		N/A	R-induced
AT2G46790	PRR9	–	–	–	–	1.85	2.28	–	3	S	ANOM	3		N/A	R-induced
AT1G09250	AIF4	–	–	–	–	–	–	–	7	S	ANOM	3		N/A	R-induced

– indicates no SSTF changes in transcript levels.

*** indicates statistically-significant 1.5-fold change (*P*-value < 0.1; used only for recategorization).

4.72 indicates statistically-significant 26.4-fold (2^4.72101552) change for the indicated comparison.

a log_2_FC of transcript levels after exposure of 3-day-old dark-grown seedlings exposed to 60 min of red (R) light.

b log_2_FC of transcript levels in 3-day-old dark-grown *pifq* mutant seedlings relative to WT.

c log_2_FC of transcript levels in 3-day-old WL-grown seedlings exposed to 30, 60, 120 or 180 min supplemental far red (FR).

d log_2_FC of transcript levels in 3-day-old white light (WL)-grown *pifS* relative to 3-day-old WL-grown WT exposed to 180 min supplemental FR.

e confirmed binding by PIF1 (1), PIF3 (3), PIF4 (4), PIF5 (5), and/or PIF7 (7).

f original categorized class (E, ES, or S).

g new class after resorting: E, ES, S, or anomalous (ANOM).

h Group 1, genes SSTF downregulated by R light **AND** SSTF downregulated in *pifq* **AND** PIF-bound (1, 3, 4, 5, and/or 7); Group 2, genes SSTF upregulated by FR light (30, 60, 120, and/or 180 min) **AND** SSTF downregulated in *pifS* **AND** PIF-bound (1, 3, 4, 5, and/or 7); Group 3, genes SSTF upregulated by FR light (30, 60, and/or 120 min) **AND** PIF-bound (PIF1, 3, 4, 5, and/or 7). .

i If described in Pfeiffer et al. (2014): Induced (Ind) or Repressed (Rep).

j rationale for inclusion in “anomalous” category.

**Figure 2:**
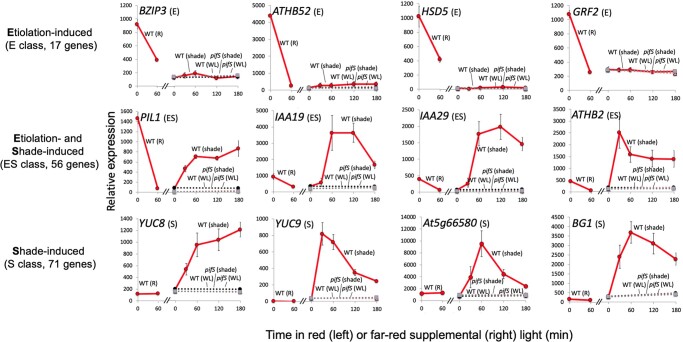
PIF-activated DTGs can be subdivided into three categories based on their responses to R light and simulated shade. Examples of transcript time course profiles for Etiolation-induced only (E) genes (*BZIP3*, *ATHB52*, *HSD5*, and *GRF2*), etiolation and shade-induced (ES) genes (*PIL1*, *IAA19*, *IAA29*, and *ATHB2*) and shade-induced only (S) genes (*YUC8*, *YUC9*, *At5g66580*, and *BG1*) class genes. Left subpanel shows the effect of 60 min R light on the transcript levels in 3-day-old dark-grown WT seedlings (solid red line). Right subpanel shows the effect of 30, 60, 120, and 180 min of FR-enriched WL or continuous WL on transcript levels in 3-day-old WL-grown seedlings (WT, FR: solid red line; *pifS*, FR: dotted black line; WL: dotted red line; *pifS*, WL: dotted gray line). Error bars indicate se from three biological replicates.

As described in Leivar et al. (2012), PIF DTGs may be broadly classified into one of three classes: re-labeled here as E, ES, and S (E for Etiolation-induced only; ES for both Etiolation- and Shade-induced; and S for Shade-induced only) (Figure 2). We therefore subdivided our combined 169 shade-light-induced and red-repressed PIF DTGs into these classes based on their patterns of expression during the D to R, and WL to shade-light transitions. Using these criteria, our initial list of 169 genes was found to contain 24 E genes, 17 ES genes, and 128 S genes (Table 1). Upon further analysis, we removed 25 genes that exhibited various anomalous expression profiles and resorted the remaining 144 genes using relaxed cutoff criteria. This resulted in a redistribution between the classes so that the final numbers of genes in each class were: 17 E genes, 56 ES genes, and 71 S genes (Table 1).

### Examination of potential epigenetic regulation of DTGs

We next tested our hypothesis that the variation in transcriptional responses of the PIF-activated DTGs to darkness and shade might be due to differences in histone tail modifications. One histone mark, H3K27me3, has already been linked to light-mediated transcriptional repression (Charron et al., 2009). Because we were focused on loci at which PIFs act as transcriptional activators, we sought to examine the levels of a histone mark associated with active transcription. One such mark, H3K4me3 is both correlated with actively transcribed genes (Bernstein et al., 2002) and inversely correlated with H3K27me3 levels (Zhang et al., 2009). We, therefore, chose to assay H3K4me3 levels at the TSS of E, ES, and S genes by ChIP-seq. We measured H3K4me3 levels in dark-grown seedlings and in WL-grown seedlings after exposure to 0, 30, 60, 120, and 180 min of simulated shade, and after 180 min of further retention in WL. We also measured H3K4me3 levels in WL-grown *pifS* seedlings after 0 and 180 min of simulated shade and after 180 min of continued WL.

**Figure 3:**
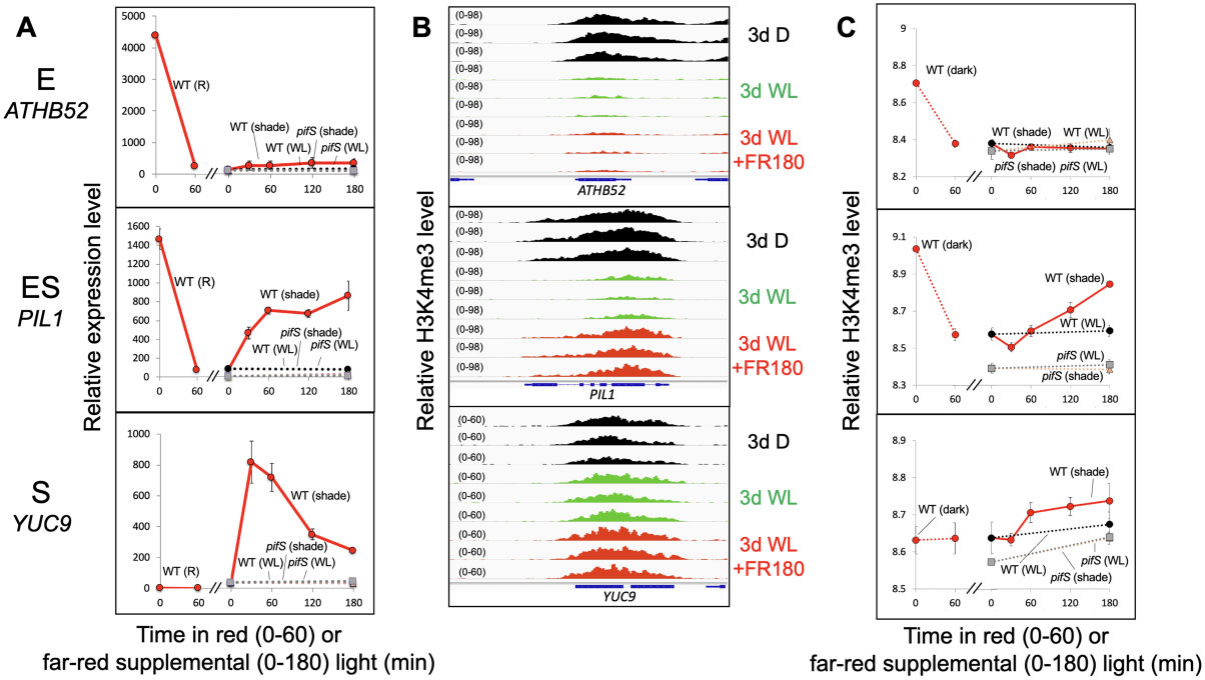
Transcript levels are broadly correlated with H3K4me3 levels for PIF DTGs belonging to etiolation-induced only (E), etiolation and shade-induced (ES) and shade-induced only (S) classes. A, Average relative transcript levels as measured by RNA-seq of *ATHB52* (E class, top), *PIL1* (ES class, middle) and *YUC9* (S class, bottom). Left subpanel shows the effect of 60 min R light on the transcript levels in 3-day-old dark-grown WT seedlings (solid red line). Right subpanel shows the effect of 30, 60, 120, and 180 min of FR-enriched WL or continuous WL on transcript levels in 3-day-old WL-grown seedlings (WT, FR: solid red line; *pifS*, FR: dotted black line; WT, WL: dotted red line; *pifS*, WL: dotted gray line). Error bars indicate se from three biological replicates. B, H3K4me3 enrichment as measured by ChIP-seq of *ATHB52* (top), *PIL1* (middle) and *YUC9* (bottom) in 3-day-old dark-grown seedlings (3 days D, black, top three tracks), 3-day-old WL-grown seedlings (3 day WL, green, middle three tracks) and 3-day-old WL-grown seedlings after 180 min of FR-enriched WL (3 day WL +FR180, red, bottom three tracks). Data from each of three biological replicates are shown. C, Average relative H3K4me3 levels of *ATHB52* (top), *PIL1* (middle), and *YUC9* (bottom). Left subpanel shows the levels in 3-day-old dark-grown seedlings and the levels in 3-day-old WL-grown seedlings (WT, connected by dashed red line to the 3-day-old WL-grown level). Right subpanel shows the effect of 30, 60, 120, and 180 min of FR-enriched WL or continuous WL on transcript levels in 3-day-old WL-grown seedlings (WT, FR: solid red line; *pifS*, FR: dotted black line; WT, WL: dotted red line; *pifS*, WL: dotted gray line). Error bars indicate SE from three biological replicates.

**Figure 4:**
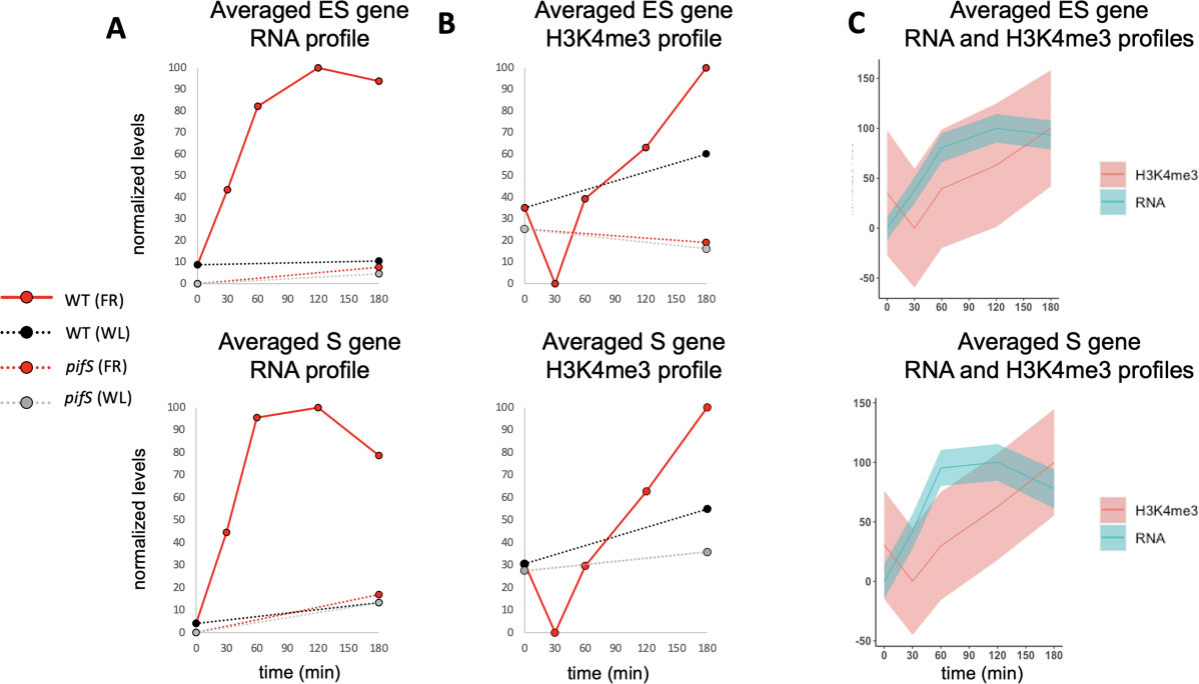
Shade-induced, PIF-dependent increases in transcription precede corresponding increases in H3K4me3 for ES class and S class genes. A, Normalized levels of the average transcript profile for all ES class genes (top) or S class genes (bottom) during 180-min shade-light (FR, red) or WL (black/gray) treatment for WT (solid red/dashed black) or *pifS* (dashed red/dashed gray) where 100 represents the maximum level and 0 represents the minimum level. B, Normalized levels of the average H3K4me3 profile for all ES class genes (top) or S class genes (bottom) during 180-min shade-light (FR, red) or WL (black/gray) treatment for WT (solid red/dashed black) or *pifS* (dashed red/dashed gray). C, Overlays of the normalized RNA (blue) and H3K4me3 (red) profiles from WT seedlings during the FR treatment. Shaded variance indicates normalized SE from three biological replicates.

As expected, H3K4me3 levels for E class genes were higher in D than in WL and simulated shade (Figure 3). On average, H3K4me3 levels for ES and S class genes increase over the course of the shade treatment and this increase is attenuated in the *pifS* mutant (Figure 4). In both classes, however, the increase only occurs after 60 min of FR, while an increase in transcript-level abundance is already visible after 30 min of FR. Both classes also exhibit a transient reduction in H3K4me3 levels after 30 min of FR. Collectively, these data indicate that the shade signal induces a transcriptional response prior to the induction of increased H3K4 trimethylation in these DTGs.

## Discussion

As a prelude to exploring the role of epigenetic factors in light/shade-regulated gene expression, we generated a set of 144 “high-confidence,” PIF-induced DTGs, that we identified by integrating our data with previously published analyses. This provided three subclasses of PIF-DTGs, displaying three contrasting patterns of transcriptional responsiveness to light and shade signals (E, ES, and S) during young seedling development. By focusing on the shade-responsiveness of these gene sets, we were able to concurrently assess whether differences in the epigenetic landscape might be associated with the observed transcriptional pattern differences, and whether comparison of the temporal patterns of shade-induced transcript and H3K4me3 changes might indicate the potential sequence of such changes.

Broadly speaking, our data are consistent with previous studies reporting that high H3K4me3 levels are correlated with actively transcribing genes. However, comparison of our integrated RNA-seq and ChIP-seq analyses over time following shade exposure, showed no clear temporal coincidence of transcript and H3K4me3 levels. On the contrary, for the shade-induced PIF DTGs, we found that, on average, transcript levels rise before their corresponding H3K4me3 levels rise (Figure 4). These results indicate that H3K4me3 plays little or no role in causing or priming the rapid, shade-induced transcriptional responsiveness of these genes. Instead, the data are more consistent with previous reports indicating that high levels of transcription from a given locus leads to trimethylation of H3K4 (Le Martelot et al., 2012; Kuang et al., 2014).

Moreover, consideration of our current findings in the context of recent advances in understanding chromatin involvement in controlling plant gene expression, suggests an intriguing possible role for H3K4me3 in shade-regulated expression through the PIF-signaling hub. Willige et al. (2021) reported that shade rapidly (within 5 min) induces the binding of PIF7 to the promoter of the *ATHB2* gene, and similarly rapidly triggers ejection of the histone variant H2A.Z, as well as increasing H3K9 acetylation (H3K9ac). These findings indicate that PIF7 occupancy of target gene promoters can shape the local chromatin status in response to shade. These changes preceded changes in gene expression, leading to the conclusion that chromatin remodeling is not a consequence of transcriptional activation. Given, first, that our data indicate, conversely to those of Willige et al. (2021), that the shade-invoked, PIF-mediated induction of target gene expression appears to precede the increases in H3K4me3 levels at those genes; and secondly, that these H3K4me3 increases are slower than both (1) the shade-induced increases in H3K9ac levels at both PIF7-binding sites and gene bodies reported by Willige et al. (2021) and (2) the light-triggered decrease of this mark in gene bodies of dark-grown seedlings observed by González-Grandío et al. (2022), it appears that H3K4me3 may be a trailing indicator of the expression status of shade-induced genes. This conclusion raises the possibility that H3K4me3 may function to stabilize the active transcriptional state of these genes, thus providing a form of transcriptional memory (Foroozani et al., 2021) as a buffer against exposure to the rapid, random fluctuations between full sunlight and shade that occur within leaf canopies, as a result of breeze-induced movement under natural conditions. The mechanism by which PIF binding activates H3K4 trimethylation at the TSS of PIF DTGs remains to be determined. However, a recent report has suggested that, at least in human cells, active transcription by RNA polymerase II is required for the deposition and persistence of H3K4me3 (Wang et al., 2022). These data, together with our results and the results of Willige et al. are consistent with a model in which PIF binding initiates H2A.Z ejection, that is followed by increases in H3K9 acetylation and transcription of PIF DTGs, with continued transcription leading to the stable accumulation of H3K4me3.

Collectively, these changes in chromatin landscape add another dimension of complexity to the multilayered network of mechanisms and pathways that regulate and intersect with the phy–PIF module. The phy family have dual photosensory and thermosensory functions, monitoring both light and temperature signals from the environment, that are then transduced through the PIFs (Leivar and Monte, 2014; Legris et al., 2016; Paik et al., 2017). In addition, the PIF family function as a signaling hub for multiple other signaling pathways, that include the core circadian oscillator, via the TIMING OF *CAB* EXPRESSION1 component and its PSEUDO-RESPONSE REGULATOR (PRR) relatives (Soy et al., 2016; Martín et al., 2018; Zhang et al., 2020), the hormones gibberellic acid, abscisic acid, jasmonic acid, ethylene, and brassinosteroids (Leivar and Monte, 2014; Paik et al., 2017), as well as interacting with the blue-light photoreceptor, cryptochrome 2 (Más et al., 2000; Pedmale et al., 2016), and numerous other factors, which together are involved in a diversity of molecular functions, that include transcriptional and posttranscriptional modulation (Wang et al., 2021), phosphorylation, ubiquitination, and degradation. Moreover, many of these light-induced interactions appear to take place in nuclear photobodies (Legris et al., 2019), functioning either as a concentrated milieu of dynamically changing, multi-component complexes, driving enhanced intermolecular interactions (Wang et al., 2021), or as foci of sequestration, as shown for PIF7 (Willige et al., 2021).

## Materials and methods

### Plant growth and phenotyping

All Arabidopsis (*A.thaliana*) seeds were stratified for 4 days at 4° before germination. Germination was induced by 3 h of incubation under 30 µmol m^−2^ s^−1^ WL at 21° followed by a 5 min saturating pulse of FR light. Seedlings were grown for 3 days at 21° in complete darkness or under 30 µmol m^−2^ s^−1^ WL (R/FR = 6–8). For FR light treatment, seedlings were grown for 3 days in WL before exposing them to simulated shade (30 µmol m^−2^ s^−1^, R/FR ∼0.3). R light was defined as 640–680 nm and FR was defined as 710–750 nm.

Hypocotyl measurements were performed on seedlings grown at 23° for 2 days in WL and either exposed to simulated shade for 4 days or kept in constant WL for 4 days. Three independent biological replicates were performed, each of which involved the plating of at least 30 seeds of each genotype all on the same plate. Plates were photographed with a high-resolution camera and hypocotyl lengths were measured via ImageJ. Mean hypocotyl length of each genotype was determined by averaging the means of the three replicates. Standard error was determined by dividing the standard deviation between all three replicas by the square root of 3. Student’s *t* test was performed for determination of *P*-values.

### RNA-seq analysis

RNA was isolated as described (Zhang et al., 2013). Total RNA was extracted from 3-day-old seedlings using a QIAshredder and RNeasy Plus Mini Kit (Qiagen, Hilden, Germany) according to manufacturer’s instructions. RNA libraries for sequencing were prepared at the Functional Genomics Laboratory at UC Berkeley using a KAPA RNA HyperPrep Kit (Roche, Basel, Switzerland) according to the manufacturer’s instructions.

RNA libraries were sequenced by the Genomic Sequencing Facility at UC Berkeley. Multiplexed RNA libraries were sequenced by 100-bp paired-end sequencing over two lanes on a HiSeq4000.

For mapping and analysis of RNA-seq experiments, reads were mapped to the Arabidopsis genome (TAIR10) by TopHat (Trapnell et al., 2009) (max intron length = 3000, inner mean distance = 200, inner distance standard deviation = 100, minimal allowed intron size = 25). Assembled reads were counted using featureCounts (Liao et al., 2014) and differential expression was determined via DESeq2 (Love et al., 2014) (log_2_FC > 1 or log_2_FC < −1; *P* < 0.05).

### Generation of PIF DTG list and subcategorization into E, ES, and S classes

To identify PIF DTGs, we first imposed strict statistically significant two-fold (SSTF) cutoffs and selected all 764 genes whose expression levels decreased in response to R light (Pfeiffer et al., 2014) and/or increased in response to shade light (this study). We then further narrowed our list to include only those genes that show a dependence on PIFs for their expression by combining the previously published RNA-seq data from the *pifq* mutant grown in darkness (Pfeiffer et al., 2014), with our RNA-seq data, obtained using the *pifqpif6pif7* mutant (*pifS*) grown in WL and exposed to 3-h shade light. We selected only those genes that were SSTF induced in WT relative to their levels in the corresponding *pif* mutant. By filtering out those genes that were not among the 764 light-responsive genes identified above, we were left with 278 PIF-dependent, light-responsive genes. Selecting only those genes that were found to be bound by one or more PIF (Hornitschek et al., 2012; Oh et al., 2012; Zhang et al., 2013; Pfeiffer et al., 2014; Chung et al., 2020) yielded 169 genes (Table 1).

We subcategorized genes into E, ES, and S classes as in Leivar et al, 2012. Class E (formerly Class L) represents genes whose dark-grown WT transcript levels are both (1) SSTF higher than those in dark-grown *pifq* and (2) SSTF repressed by the initial R light signal in WT. Although some Class E genes show a degree of re-induction in the shade, this is weaker (i.e. non-SSTF), and the PIF dependency is less, than initially in the dark (Figure 2). Conversely, Class S (formerly Class R) represents genes that do display SSTF induction by shade light, as well as PIF-dependent SSTF induction in the shade, but that do not exhibit a SSTF response to either: (1) the PIFs in dark-grown seedlings or (2) R light exposure (Figure 2). Finally, Class ES (formerly Class M) represents those genes that display SSTF, mutually converse responsiveness to the onset of the light and shade-light signals, respectively, as well as PIF-dependent SSTF induction, both in the dark and in the shade light (Figure 2).

A subset of these E, ES, and S class genes exhibited anomalous transcription profiles. We manually removed these 25 genes because they were either highly expressed in WL (6 genes), were induced, rather than repressed, by R light (16 genes), were lowly expressed (1 gene) or were otherwise likely to be artifactual (2 genes). The remaining 144 PIF DTGs were then resorted using relaxed cutoffs. Of the nonanomalous genes first categorized as S class, 38 showed an R-dependent reduction (*P* < 0.1) in transcript levels but were excluded from the ES class because they did not show an SSTF reduction in dark-grown *pifq* mutant relative to WT. These genes were reclassified as ES. Two E class genes were also reclassified as ES genes because they exhibited a statistically significant upregulation in response to FR despite not being SSTF downregulated in the *pifS* mutant. Ultimately, we were left with 17 E genes, 56 ES genes, and 71 S genes (Table 1).

### H3K4me3 ChIP-seq analysis

DNA libraries were sequenced by the Genomic Sequencing Facility at UC Berkeley. The multiplexed DNA libraries were sequenced by 50-bp single-end sequencing over two lanes on a HiSeq4000.

For mapping and analysis of ChIP-seq experiments, reads were mapped to the Arabidopsis genome (TAIR10) by BowTie2 (Langmead and Salzberg, 2012) and uniquely mapping reads were first used to call peaks using BayesPeak (Spyrou et al., 2009) (Bioconductor 3.6; binsize = 300, peaks with a PP > 0.999 in all three biological replicates) or model-based analysis of ChIP-seq (MACS) (Zhang et al., 2008). H3K4me3 peaks calculated using BayesPeak and MACS2 could only be unambiguously assigned to the TSS of 102 of the 144 E, ES, and S class genes. To ensure consistency in analysis, we therefore manually assigned peaks to all of the PIF DTGs by creating 300-bp windows centered on the TSS.

To quantify the H3K4me3 peaks and measure differences between time points we used DiffBind (Ross-Innes et al., 2012) and DESeq2 (Love et al., 2014). Because the changes in magnitude of H3K4me3 levels were far smaller than the changes in transcript abundance, we used DESeq2 to calculate variance-stabilizing transformations across the time course experiment for both H3K4me3 levels and transcript levels. This enabled comparison of relative changes in H3K4me3 levels to the corresponding changes in transcription for a given gene or class of genes.

### Accession numbers

Sequence data from this article can be found in the National Center for Biotechnology Information (NCBI) data libraries under BioProject ID number PRJNA839161.

## Notes

R.H.C. and P.H.Q. designed the research. R.H.C., J.D., and Y.Z. performed the research. R.H.C., J.D., Y.Z., and P.H.Q. analyzed the data. R.H.C. and P.H.Q. wrote the paper.

The author responsible for distribution of materials integral to the findings presented in this article in accordance with the policy described in the Instructions for Authors (https://academic.oup.com/plphys/pages/general-instructions) is Robert H. Calderon (robert.calderon@umu.se).
